# Supplementary material for: Transcriptomic Differences Underlying the Activin-A Induced Large Osteoclast Formation in Both Healthy Control and Fibrodysplasia Ossificans Progressiva Osteoclasts
Source: Int J Mol Sci. 2023 Apr 6;24(7):6822. doi: 10.3390/ijms24076822 (PMC10095588; doi:10.3390/ijms24076822)
Supplement: Supplementary file 1 [file ijms-24-06822-s001.zip › ijms-2228118-supplementary.pdf]

## Supplementary Table S1. RNA Quality Control

| Control | | FOP | |
|---|---|---|---|
| **Sample** | **RIN$^e$ value** | **Sample** | **RIN$^e$ value** |
| Con1−ActA | 8.3 | FOP1−ActA | 8.6 |
| Con1+ActA | 8.0 | FOP1+ActA | 9.3 |
| Con2−ActA | 7.9 | FOP2−ActA | 1.8 |
| Con2+ActA | 8.4 | FOP2+ActA | 9.0 |
| Con3−ActA | 7.8 | FOP3−ActA | 8.0 |
| Con3+ActA | 1.0 | FOP3+ActA | 8.5 |
| Con4−ActA | 8.4 | FOP4−ActA | 8.4 |
| Con4+ActA | 9.1 | FOP4+ActA | 8.9 |
| Con5−ActA | 8.7 | FOP5−ActA | 7.6 |
| Con5+ActA | 8.2 | FOP5+ActA | 7.6 |
| Con6−ActA | 8.3 | FOP6−ActA | 8.2 |
| Con6+ActA | 9.0 | FOP6+ActA | 9.0 |

**Supplementary Table S1**. RNA integrity number (RIN) values were determined on RNA isolated from CD14-positive monocytes from a group of FOP patients and a group of healthy controls treated with (+ActA) or without (−ActA) Activin-A.

## Supplementary Table S2. Most significantly upregulated genes

| ENSEMBL ID | Gene name | *p*(adj)-value | Log2 fold change |
|---|---|---|---|
| ENSG00000173391 | *OLR1* | 3,79E-77 | 4,30 |
| ENSG00000204252 | *HLA-DOA* | 1,76E-53 | 4,20 |
| ENSG00000179583 | *CIITA* | 2,34E-53 | 3,15 |
| ENSG00000011201 | *ANOS1* | 5,05E-46 | 2,12 |
| ENSG00000197093 | *GAL3ST4* | 4,42E-45 | 5,61 |
| ENSG00000166033 | *HTRA1* | 3,53E-42 | 2,17 |
| ENSG00000072422 | *RHOBTB1* | 5,50E-41 | 2,07 |
| ENSG00000151117 | *TMEM86A* | 7,76E-35 | 2,26 |
| ENSG00000102996 | *MMP15* | 3,70E-34 | 2,20 |
| ENSG00000134508 | *CABLES1* | 8,39E-34 | 4,04 |
| ENSG00000166927 | *MS4A7* | 4,61E-33 | 3,05 |
| ENSG00000116774 | *OLFML3* | 1,40E-32 | 5,21 |
| ENSG00000198286 | *CARD11* | 2,95E-32 | 3,01 |
| ENSG00000124225 | *PMEPA1* | 6,07E-32 | 2,92 |
| ENSG00000169116 | *PARM1* | 4,71E-31 | 4,59 |
| ENSG00000182985 | *CADM1* | 4,66E-30 | 2,70 |

| | | | |
|---|---|---|---|
| ENSG00000182578 | *CSF1R* | 1,04E-29 | 2,01 |
| ENSG00000145284 | *SCD5* | 5,80E-28 | 3,60 |
| ENSG00000102221 | *JADE3* | 2,35E-26 | 2,41 |
| ENSG00000232629 | *HLA-DQB2* | 1,11E-25 | 2,65 |
| ENSG00000179715 | *PCED1B* | 3,20E-25 | 2,99 |
| ENSG00000184588 | *PDE4B* | 3,74E-25 | 2,54 |
| ENSG00000163995 | *ABLIM2* | 4,94E-25 | 2,95 |
| ENSG00000137642 | *SORL1* | 7,87E-25 | 2,72 |
| ENSG00000188070 | *C11ORF95* | 8,51E-25 | 2,13 |
| ENSG00000120708 | *TGFBI* | 1,00E-24 | 3,31 |
| ENSG00000196664 | *TLR7* | 2,77E-24 | 3,09 |
| ENSG00000134042 | *MRO* | 6,46E-24 | 3,48 |
| ENSG00000204174 | *NPY4R* | 6,46E-24 | 5,05 |
| ENSG00000164176 | *EDIL3* | 7,46E-24 | 4,32 |
| ENSG00000148400 | *NOTCH1* | 9,59E-24 | 2,32 |
| ENSG00000164171 | *ITGA2* | 1,98E-23 | 4,22 |
| ENSG00000213420 | *GPC2* | 4,49E-23 | 2,99 |
| ENSG00000223865 | *HLA-DPB1* | 4,73E-23 | 2,54 |
| ENSG00000273415 | *LINC02725* | 8,63E-23 | 2,67 |
| ENSG00000104059 | *FAM189A1* | 2,04E-22 | 4,10 |
| ENSG00000164037 | *SLC9B1* | 2,15E-22 | 3,49 |
| ENSG00000106976 | *DNM1* | 5,99E-22 | 3,56 |
| ENSG00000183019 | *MCEMP1* | 6,24E-22 | 4,36 |

**Supplementary Table S2**. Most significantly upregulated genes present in the 100 most significantly differentially expressed genes sorted on p(adj) value

## Supplementary Table S3. Most significantly downregulated genes

| ENSEMBL ID | Gene name | *p*(adj)-value | Log2 fold change |
|---|---|---|---|
| ENSG00000271856 | *LINC01215* | 9,23E-54 | -4,18 |
| ENSG00000174370 | *C11ORF45* | 1,53E-39 | -3,01 |
| ENSG00000180509 | *KCNE1* | 2,46E-39 | -2,36 |
| ENSG00000151704 | *KCNJ1* | 5,09E-38 | -3,70 |
| ENSG00000118971 | *CCND2* | 1,44E-37 | -2,79 |

| | | | |
|---|---|---|---|
| ENSG00000111859 | *NEDD9* | 1,10E-34 | -3,66 |
| ENSG00000168350 | *DEGS2* | 3,37E-34 | -3,14 |
| ENSG00000036672 | *USP2* | 9,74E-34 | -2,33 |
| ENSG00000075340 | *ADD2* | 1,23E-33 | -4,20 |
| ENSG00000243742 | *RPLP0P2* | 7,24E-33 | -2,93 |
| ENSG00000117586 | *TNFSF4* | 9,50E-33 | -2,44 |
| ENSG00000181634 | *TNFSF15* | 1,40E-32 | -3,15 |
| ENSG00000245248 | *USP2-AS1* | 6,07E-32 | -3,59 |
| ENSG00000166501 | *PRKCB* | 6,27E-32 | -2,84 |
| ENSG00000286872 | *AC024270.4* | 1,31E-30 | -2,53 |
| ENSG00000170485 | *NPAS2* | 2,27E-30 | -4,25 |
| ENSG00000129451 | *KLK10* | 4,45E-30 | -4,48 |
| ENSG00000147509 | *RGS20* | 5,60E-30 | -2,96 |
| ENSG00000147168 | *IL2RG* | 5,77E-30 | -2,13 |
| ENSG00000203685 | *C1ORF95* | 9,06E-30 | -3,77 |
| ENSG00000256508 | *MRGPRF-AS1* | 5,70E-29 | -4,18 |
| ENSG00000254740 | *AP003396.3* | 3,75E-28 | -3,66 |
| ENSG00000156011 | *PSD3* | 5,49E-28 | -2,73 |
| ENSG00000152413 | *HOMER1* | 5,80E-28 | -2,92 |
| ENSG00000276289 | *KCNE1B* | 7,98E-28 | -2,47 |
| ENSG00000232453 | *LOC105378753* | 3,52E-27 | -3,99 |
| ENSG00000073712 | *FERMT2* | 5,46E-27 | -2,95 |
| ENSG00000157470 | *FAM81A* | 6,61E-27 | -3,26 |
| ENSG00000147231 | *CXORF57* | 8,03E-27 | -2,44 |
| ENSG00000172575 | *RASGRP1* | 1,13E-26 | -2,15 |
| ENSG00000232258 | *TMEM114* | 4,20E-26 | -3,18 |
| ENSG00000162551 | *ALPL* | 5,17E-26 | -4,24 |
| ENSG00000119866 | *BCL11A* | 9,93E-26 | -2,59 |
| ENSG00000175175 | *PPM1E* | 1,08E-25 | -2,85 |
| ENSG00000077782 | *FGFR1* | 2,20E-25 | -2,34 |
| ENSG00000176928 | *GCNT4* | 3,18E-25 | -2,80 |
| ENSG00000162896 | *PIGR* | 6,60E-25 | -3,61 |
| ENSG00000175356 | *SCUBE2* | 7,21E-25 | -3,45 |

| | | | |
|---|---|---|---|
| ENSG00000155886 | *SLC24A2* | 1,16E-24 | -5,53 |
| ENSG00000228486 | *LINC01125* | 1,36E-24 | -3,05 |
| ENSG00000188613 | *NANOS1* | 1,57E-24 | -3,20 |
| ENSG00000163803 | *PLB1* | 1,89E-24 | -2,10 |
| ENSG00000117245 | *KIF17* | 2,59E-24 | -2,79 |
| ENSG00000185022 | *MAFF* | 4,36E-24 | -2,33 |
| ENSG00000107736 | *CDH23* | 4,64E-24 | -3,09 |
| ENSG00000234572 | *LOC101927438* | 4,71E-24 | -4,14 |
| ENSG00000011347 | *SYT7* | 4,83E-24 | -3,97 |
| ENSG00000263325 | *AC003965.2* | 5,01E-24 | -3,52 |
| ENSG00000111371 | *SLC38A1* | 9,23E-24 | -4,07 |
| ENSG00000166949 | *SMAD3* | 1,59E-23 | -2,96 |
| ENSG00000284240 | *LINC02801* | 1,67E-23 | -3,82 |
| ENSG00000076706 | *MCAM* | 4,74E-23 | -4,13 |
| ENSG00000180720 | *CHRM4* | 7,51E-23 | -4,52 |
| ENSG00000000938 | *FGR* | 1,08E-22 | -2,16 |
| ENSG00000135525 | *MAP7* | 2,00E-22 | -2,58 |
| ENSG00000162894 | *FCMR* | 2,15E-22 | -3,11 |
| ENSG00000214212 | *C19ORF38* | 2,23E-22 | -2,15 |
| ENSG00000102003 | *SYP* | 2,56E-22 | -3,18 |
| ENSG00000155893 | *PXYLP1* | 2,58E-22 | -2,07 |
| ENSG00000196395 | *AC244505.1* | 4,78E-22 | -2,75 |
| ENSG00000172935 | *MRGPRF* | 4,81E-22 | -3,33 |

**Supplementary Table S3**. Most significantly downregulated genes present in the 100 most significantly differentially expressed genes sorted on p(adj) value

## Supplementary Table S4. Upregulated genes per cellular process during cell fusion

| Differentiation | Actin Organization | Chemotaxis | Adhesion | Fusion |
|---|---|---|---|---|
| *APLP1* | *ACTN2* | *ANGPT1* | *ADAM19* | *ACTN2* |
| *AXL* | *ASAP3* | *CALCR* | *CARD11* | *ADAM12* |

| | | | | |
|---|---|---|---|---|
| *CALCR* | *CADM1* | *CCL18* | *CD244* | *ADCY1* |
| *CNGB1* | *CDC42EP5* | *CMKLR1* | *CDH5* | *ADGRB1* |
| *CSF1R* | *CDH5* | *CSF1R* | *EFNB3* | *CHRNA6* |
| *DNM1* | *CLDN4* | *CXCL12* | *GRAP2* | *CST3* |
| *F2* | *CSF1R* | *CXCR3* | *HLA-DPA1* | *CXCL1* |
| *GAS6* | *CTNNA2* | *EXT1* | *HLA-DPB1* | *CXCL12* |
| *GIPR* | *CTNND2* | *FFAR4* | *HLA-DRA* | *DGKI* |
| *HLA-DRA* | *CXCL12* | *FLT4* | *IGF1* | *DNM1* |
| *IL2RA* | *DGKI* | *GAS6* | *IL2RA* | *EHD2* |
| *IL6* | *EPHA2* | *GPBAR1* | *IL6* | *F5* |
| *IL7R* | *ESPNL* | *IL6* | *IL7R* | *FPR1* |
| *ITGB3* | *EXT1* | *ITGA2* | *KIF26B* | *FUCA1* |
| *PDE4B* | *F2* | *ITGB3* | *NOTCH1* | *GAS6* |
| *PPEF1* | *F2R* | *JAM3* | *PLEKHA7* | *HGF* |
| *RGS10* | *F2RL2* | *NOTCH1* | *SPTA1* | *HPSE* |
| *RGS4* | *GJA1* | *P2RY6* | *WNT5A* | *IGF1* |
| *ROR2* | *GPBAR1* | *PDGFD* | | *ITGB3* |
| *SLC9B2* | *GPR35* | *PTN* | | *LCN2* |
| | *GRIP1* | *ROR2* | | *LRRC7* |
| | *ID1* | *S1PR1* | | *MCEMP1* |
| | *IGF1* | *SELL* | | *NCAM1* |
| | *IL6* | *THBS1* | | *NOTCH1* |
| | *ITGB3* | *TRPV4* | | *OLR1* |
| | *JAM3* | *WNT5A* | | *PFN2* |
| | *MAP1A* | | | *PRSS12* |
| | *MARCKSL1* | | | *RAB3B* |
| | *MARVELD3* | | | *RETN* |
| | *MID1* | | | *RNASE2* |
| | *MYO1B* | | | *RNASE3* |
| | *NET1* | | | *SCGB3A1* |
| | *NLGN4X* | | | *SELL* |
| | *NOTCH1* | | | *SYT3* |
| | *P2RY8* | | | *TCN1* |

| | |
|---|---|
| *PEAK3* | *THBS1* |
| *PFN2* | |
| *PLEKHA7* | |
| *PMP22* | |
| *PROX1* | |
| *PTN* | |
| *RASAL1* | |
| *RGS4* | |
| *RHOBTB1* | |
| *S1PR1* | |
| *SEMA3E* | |
| *SEMA4D* | |
| *SLC39A12* | |
| *SPTA1* | |
| *SYT3* | |
| *TRPV4* | |
| *WASF3* | |
| *WNT5A* | |
| *ZNF664-RFLNA* | |

**Supplementary Table S4**. Significantly upregulated genes with cut- off values of p(adj)<0.01 and Log2Fold change ≥ 2, divided into the different stages of cell fusion they are associated with.

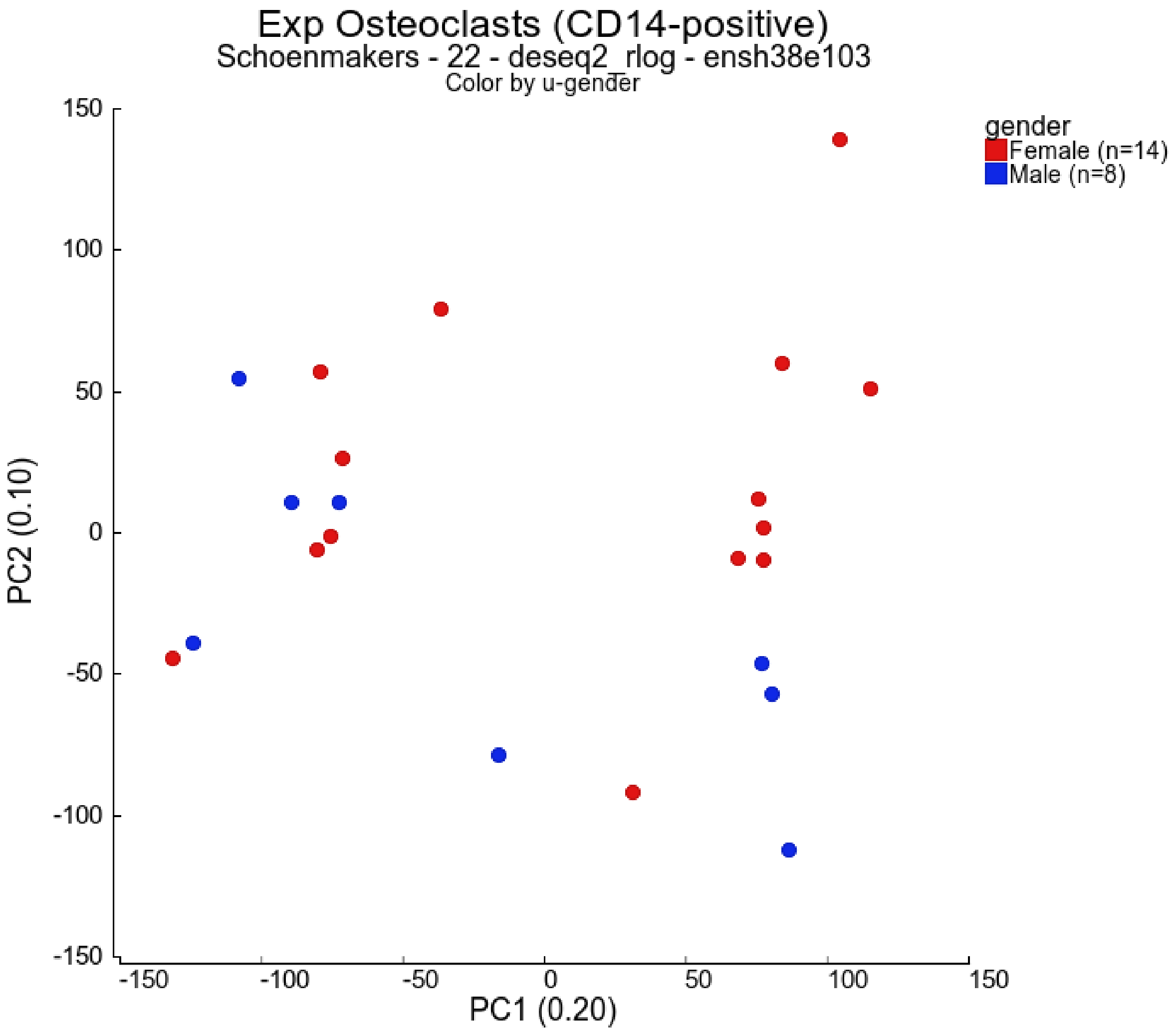


Figure S1 **PCA plot depicting inter-gender variability. Control and FOP female donors (both – and + Activin-A) depicted in red, Control and FOP male donors (both – and + Activin-A) depicted in blue.**
